# Bi-linear mechanical property determination of acellular human patellar tendon grafts for use in anterior cruciate ligament replacement

**DOI:** 10.1016/j.jbiomech.2016.03.041

**Published:** 2016-06-14

**Authors:** Anthony Herbert, Christopher Brown, Paul Rooney, John Kearney, Eileen Ingham, John Fisher

**Affiliations:** a (IMBE) Institute of Medical and Biological Engineering, School of Mechanical Engineering, University of Leeds, Leeds, UK; b IMBE, Faculty of Biomedical Sciences, University of Leeds, Leeds, UK; c NHS Blood & Transplant Services, Speke, Liverpool, UK

**Keywords:** Acellular biological scaffold, ACL replacement, Patellar tendon

## Abstract

Anterior cruciate ligament rupture is rising in its prevalence amongst the young and those with physically active lifestyles. Acellular human patellar tendon (PT) grafts offer a promising restoration solution, returning knee joint stability and overcoming some of the current disadvantages of autologous or allogeneic grafts. However, it is necessary to ensure that the decellularisation bio-processes involved do not cause structural changes in the microstructure of the tendon tissue that may adversely affect the mechanical properties, particularly with respect to the physiological range of loading.

Sixteen cadaveric human PT grafts were sourced and processed from eight donors, with full ethical approval and consent for use in research. Eight specimens were allocated for decellularisation, while the remaining eight contralateral specimens were used as native controls. Testing consisted of 12 preconditioning cycles followed by uniaxial extension until failure occurred. Stress–strain data was then fitted to a bi-linear model using least squares regression by a custom-written Matlab script. The elastic moduli for the toe region and linear region of each specimen were determined, in addition to the transition point co-ordinates and strain energy density for increasing strain. No significant differences were found between groups for all of the parameters investigated. Hence, the shape and magnitude of the stress–strain profile was found to be the same for both groups throughout loading.

The results of this study indicated that decellularisation appeared to have no effect on the material properties of human PT grafts under quasistatic conditions. Therefore, acellular human PT grafts can offer a viable additional solution for ACL replacement compared to current autologous and allogeneic treatment options.

## 1 Introduction

The anterior cruciate ligament (ACL) plays a crucial role in maintaining joint conformity and stability in the knee by restricting anterior displacement of the tibia relative to the femur (Laurencin and Freeman, 2005, Samuelsson et al., 2009). However, injury to the ACL is commonplace, particularly amongst athletes and an increasingly more physically active population. If left untreated, ACL injuries can lead to meniscus damage and degenerative changes such as osteoarthritis (Corry et al., 1999, Spindler and Wright, 2008), causing further pain and impairment of the joint.

The most common surgical solution at present is to replace the damaged ACL with autograft tissue such as hamstring (semitendinosus and gracilis) tendon or patellar tendon grafts. Although hamstring tendon grafts have gained more recent popularity, owed in part to fewer donor-site complications (Samuelsson et al., 2009), bone–patellar tendon–bone grafts have been considered by some to be the “gold standard” of ACL replacement (Woods and Gratzer, 2005) and can provide bone to bone apposition at fixation sites for more rapid integration. Furthermore, recent Norwegian and Danish registry studies indicate that patellar tendon autografts have a reduced risk of revision compared to hamstring tendon autografts (Persson et al., 2014, Rahr-Wagner et al., 2014). All autografts result in some donor site morbidity and rehabilitation. Allografts provide an attractive option as they eliminate the need to harvest any autologous material whilst reducing the overall surgical time as well as avoiding donor site morbidity. However, these too have intrinsic disadvantages such as the possibility of adverse immunological reactions (Prokopis and Schepsis, 1999). More fundamentally, both autologous and cryopreserved allogeneic graft choices suffer from the concern of preserving cell vitally. Once transplanted, it is unlikely that native cells remain vital and this can cause progressive degradation of the graft and its subsequent mechanical performance. This is because the rate of tissue degradation (as a result of necrosis) typically exceeds that of cellular in-growth and constructive remodelling (Mcfarland, 1993, Corsetti and Jackson, 1996).

Tissue engineering offers a promising solution to replacement of the ruptured ACL. The ideal tissue engineered tendon scaffold should possess similar mechanical properties to native tendon, be biocompatible, composed of a biodegradable material and provide a supportive environment for cell ingrowth (Pridgen et al., 2011). Synthetic or collagen Type I based scaffolds can be manufactured and used as platform to develop substitute tissues (Laurencin and Freeman, 2005, Petrigliano et al., 2006). However, these usually fail to provide the multi-scale hierarchical matrix architectures present in the native ACL or autologous/allogeneic grafts. Biological scaffolds produced by decellularisation of native tissues have the advantage of providing this complex hierarchical matrix and, in doing so, reproduce closely the specific biomechanical and biological functions of the tissue in question. Hence, an acellular tendon/ligament graft may be ideally positioned to replace the native ACL without any of the current disadvantages of otherwise tissue engineered or autologous/allogeneic grafts.

Decellularisation treatments are multi-faceted, vary considerably between different processes and may involve many lengthy treatment steps. Some of these bio-processes may cause undesirable structural changes to the ECM of tissues and, by association, their mechanical properties (Gilbert et al., 2006, Crapo et al., 2011). Thus, in the case of an acellular graft for ACL replacement, it is of paramount importance to ensure that the properties of the tissue are not affected to the extent of reducing their biomechanical performance and longevity. Previously, we investigated acellular porcine super flexor tendon (pSFT) as a possible graft for ACL replacement and found that decellularisation caused the tissue to become more extensible in the toe region of loading (Herbert et al., 2015). This was deemed to be an effect of the treatment process altering the crimping pattern of collagen fibres. These changes are acceptable however, as they are unlikely to detract from the tissue providing sufficient stability and support when deployed in graft format.

In this study, we determined the material properties of acellular human patellar tendon (PT) grafts with a view to investigating the decellularisation process from a biomechanical perspective. It was hypothesised that the biomechanical changes found to occur in the pSFT as a result of the decellularisation process may also be evident in decellularised human PT grafts, and therefore it was necessary to determine the extent of these changes in this graft material.

## 2 Materials and methods

### 2.1 Tissue sourcing and preparation

Sixteen patella–tendon–tibia units were sourced from eight donors (six males, two females, mean 56.25 years old, range 45–69 years old), supplied by NHS Blood & Transplant Services (Speke, Liverpool, UK), with full ethical approval and consent for use in research. The central third of the patellar and tibial bone extremities were processed into rectangular blocks of approximately 10×10×30 mm and the central tendon element was trimmed to conform to the width of these bone sections. The bone extremities were preserved and processed in this manner in order to provide fixation points for future mechanical testing. In order to minimise left/right leg selection bias, four right and four left PT articles were chosen from random donors and allocated for decellularisation. The remaining articles served as an untreated, native control group. All specimens were then frozen at −40 °C during storage prior to further use.

### 2.2 Decellularisation

Decellularisation was achieved using an adaption of a previously used protocol developed originally for the meniscus (Stapleton et al., 2008). This consisted of subjecting specimens to three freeze/thaw cycles, two of which were followed by 10 min immersion in a sonicating bath, two 10 min cycles of centrifugation in phosphate buffered saline (PBS [MP Biomedical LLP]) at 1900*g* and then cycled through hypotonic buffer (50 mM Tris pH 8 [Fisher Scientific]) plus aprotinin (10 KIU ml^−1^ [NHS Supplies, Leeds, UK]) for 24 h, sodium dodecyl sulphate (0.1% (w/v) SDS [Sigma]) in hypotonic buffer plus aprotinin (10 KIU ml^−1^ [NHS Supplies, Leeds, UK]) for 24 h twice with agitation. Specimens were washed in PBS three times prior to two cycles of incubation in Benzonase (1 U ml^−1^ [Merck]) in 50 mM Tris–HCl, 10 mM MgCl_2_, pH 7.5 for 3 h at 37 °C with gentle agitation. Tissue was then washed in hypertonic buffer (1.5 M NaCl in 0.05 M Tris–HCl, pH 7.6) prior to washing in PBS and sterilisation in peracetic acid (0.1% (w/v) [Sigma]), before final PBS washes. All treatment steps were performed in 125 ml wash volumes in 150 ml sealed laboratory pots.

### 2.3 Biomechanical testing

**Fig. 1:**
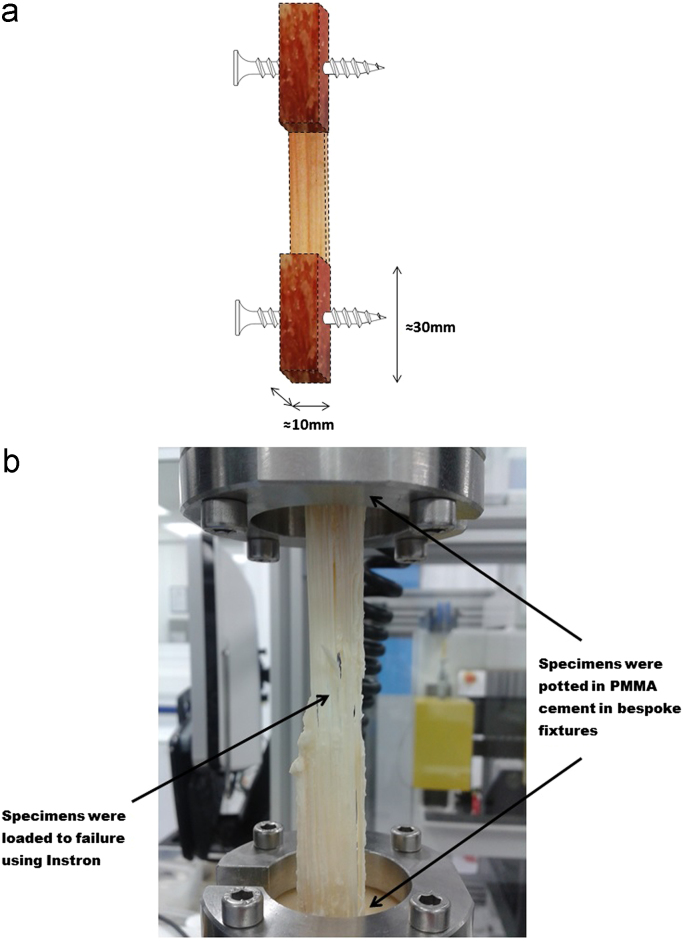
(a) Human PT specimens were processed to have 10×10×30 mm rectangular blocks of bone at each extremity, with a screw placed in each bone block to facilitate better fixation, (b) the bone blocks were then potted in PMMA cement in bespoke fixtures and mounted to an Instron where they were loaded until failure occurred.

The width and length of each patellar tendon were determined by calculating the average of three measurements with digital Vernier calipers. This method of measurement was deemed acceptable as it has been shown to produce results comparable to laser micrometre systems in similar tissues (Woo et al., 1990). Thickness was measured in a similar fashion using a thickness gauge which applied a constant force of 0.65 N. To aid fixation, screws were placed transversely through both the patellar and tibial bone sections in the coronal plane, perpendicular to the tendons (Fig. 1a). These sections were then potted using poly-methyl methacrylate cement (WHW Plastics, Hull, UK) in bespoke fixtures. The fixtures had been manufactured in such a manner to allow for subsequent attachment to an Instron 3365 uniaxial testing system with 5 kN load cell (Instron, Bucks, UK) to load the specimens to failure (Fig. 1b). During the potting process, the soft tissues were isolated and protected from thermal injury by wrapping them in PBS soaked filter paper. This was removed immediately prior to mechanical testing.

Testing consisted of 12 preconditioning cycles between 0 and 50 N at a strain rate of 0.01 s^−1^ to ensure the collagen fibres within the specimens were fully aligned to the axis of loading. This was then followed by an extension ramp to failure at the same strain rate. Such quasi-static loading conditions were chosen over more rapid physiological loading rates in order to limit the viscous contribution of the tissues to the mechanics. Hence, the tissues solid component (principally collagen Type I) was investigated alone with a view to decellularisation effects. Data was recorded at a frequency of 10 Hz. Engineering stress (*σ*) was calculated by dividing the force recorded by the load cell by the original cross-sectional area (width×thickness) of the ligament substance, whereas engineering strain (*ε*) was determined by dividing the crosshead displacement by the original length of the ligament substance.

Stress–strain data was then fitted to the following bi-linear model using non-linear least squares regression with a custom written Matlab script (Matlab (R2014a));

$$\;\sigma =E_{0}.\epsilon \;\;\;\mathrm{for}\;\epsilon \leq \epsilon_{⁎}$$

$$\sigma =E_{1}.\epsilon +c\;\mathrm{for}\;\epsilon >\epsilon_{⁎}$$

where *E*_0_ and *E*_1_ are the moduli of the toe and linear region respectively, *ε*_⁎_ is the strain at which the two linear elements intersect and *c* is a constant to be determined. Similar bi-linear constitutive models have previously been used for biological tissues (Elliott and Setton, 2001, Lynch et al., 2003, Chandrashekar et al., 2008).

**Fig. 2:**
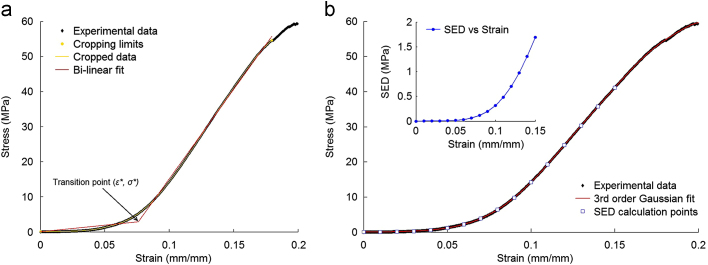
(a) Stress–strain data was fitted to a bi-linear model using a custom written Matlab programme, (b) SED was calculated with increasing strain up to 0.15. This was achieved by fitting the stress–strain data to a 3rd order Gaussian function and integrating the resulting function at increments of 0.01 strain.

Hence, in addition to elastic moduli representing the toe region and linear region, the transition point (*ε*_⁎_, *σ*_⁎_) between the toe region and the linear region was also determined, where *ε*_⁎_ is the transition strain and *σ*_⁎_ is the transition stress (Fig. 2a). The stress and strain of the ligament at failure were not determined as mid-substance failure occurred in only half the specimens tested. However, the load and extension at failure were recorded, in addition to the mechanisms of failure, as indicators of structural performance.

The strain energy density (SED) as a function of strain was also determined. This allowed for quantification of the ability of native and decellularised materials to absorb energy within the toe region through to the linear region, but critically it represents the cumulative growth in the area under the stress–strain profiles with incremental strain, and so is a useful indicator to gauge differences in the mechanical performance between the groups.

For each specimen, the following 3rd order Gaussian function was fitted (*r*^2^>0.99) to the stress–strain data up to the failure point using non-linear least squares regression:

$$\sigma \left(\epsilon \right)=\overset{3}{\underset{i=1}{\sum }}a_{i}.\exp^{\left(-{\left(\frac{\epsilon -b_{i}}{c_{i}}\right)}^{2}\right)}$$

where *a*_*i*_, *b*_*i*_, and *c*_*i*_ are constants to be determined. This has successfully been used in the fitting of stress–strain data for porcine super flexor tendon (Herbert et al., 2015).

This was then integrated and evaluated between the limits of 0 and incremental strains of 0.01 until 0.15 strain was reached. 0.15 was chosen as the terminal limit as it represented a strain value approximately half-way into the linear region. This procedure is illustrated in Fig. 2b. Hence, the SED for native and decellularised groups was assessed throughout the toe region and deep into the linear region, accounting for any strains likely to be encountered during physiological loading.

### 2.4 Statistical analyses

Student׳s *t*-tests were employed to investigate significance between anthropometrical and biomechanical parameters in native and decellularised groups. Student׳s *t*-tests were also used to determine whether significance existed between both groups for SED at each level of strain investigated. In all cases, a *p*-value of <0.05 was considered to be statistically significant.

## 3 Results

The results determined for the gross measurements, moduli, transition point coordinates in addition to the load and extension at failure are presented in Table 1. No significant differences (*p*>0.05; Student׳s *t*-tests) were found between native and decellularised PT groups for any of the parameters investigated.

**Table 1:** Results of the toe region modulus (*E*_*o*_), linear region modulus (*E*_*1*_), transition coordinates (*ε*_⁎_ – transition strain, *σ*_⁎_ – transition stress), extension at failure (*δ*_*FAIL*_), load at failure (*P*_*FAIL*_) and failure mechanisms for all specimens tested. Specimens are identified by the donor number, gender and leg from which the specimen is harvested from (i.e. D1-F-R indicates donor 1, female, right leg). No significant differences were found between native and decellularised (D-C) PT groups for any of the parameters investigated (Student׳s *t*-tests; *p*<0.05).

*Group*	*Specimen*	*E*_*0*_ (MPa)	*E*_*1*_ (MPa)	*ε*_⁎_ (mm/mm)	*σ*_⁎_ (MPa)	*δ*_*FAIL*_ (mm)		*P*_*FAIL*_ (N)	*Failure Mechanism*	
Native	D1-F-R	36.9	319	0.09	3.4	11.3		1790	Tibial Bone Avulsion	
	D2-M-L	47.9	415	0.07	3.1	8.6		1772	Tibial Bone Rupture	
	D3-M-R	70.2	509	0.06	4.3	8.1		2439	Tibial Bone Avulsion	
	D4-M-L	50.4	245	0.08	4.2	7.7		852	Tibial Bone Rupture	
	D5-M-R	43.3	407	0.10	4.2	10.6		1762	Midsubstance Failure	
	D6-M-R	63.9	470	0.07	4.6	6.6		1559	Tibial Bone Rupture	
	D7-F-L	70.9	351	0.03	2.4	11.2		3141	Patellar Bone Avulsion	
	D8-M-L	48.7	360	0.09	4.3	9.3		1796	Midsubstance Failure	
	Mean	54.0	384	0.07	3.8	9.2		1889		
	SD	12.7	84	0.02	0.8	1.7		666		
	95% CI	10.6	70	0.02	0.6	1.5		557		
	D1-F-L	34.3	275	0.13	4.5	13.0		2054	Midsubstance Failure	
	D2-M-R	67.0	318	0.05	3.0	8.1		1610	Tibial Bone Avulsion	
D-C	D3-M-L	51.3	380	0.05	2.7	8.3		2113	Tibial Bone Avulsion	
	D4-M-R	37.3	511	0.08	2.8	9.2		2557	Midsubstance Failure	
	D5-M-L	35.7	531	0.14	4.9	13.0		1584	Tibial Bone Avulsion	
	D6-M-L	49.8	418	0.08	4.0	8.7		2386	Midsubstance Failure	
	D7-F-R	81.8	477	0.08	6.1	9.9		2680	Midsubstance Failure	
	D8-M-R	70.7	295	0.04	2.6	6.2		1317	Midsubstance Failure	
	Mean	53.5	401	0.08	3.8	9.5		2038		
	SD	17.9	100	0.04	1.3	2.4		495		
	95% CI	15.0	83	0.03	1.1	2.0	414			

**Fig. 3:**
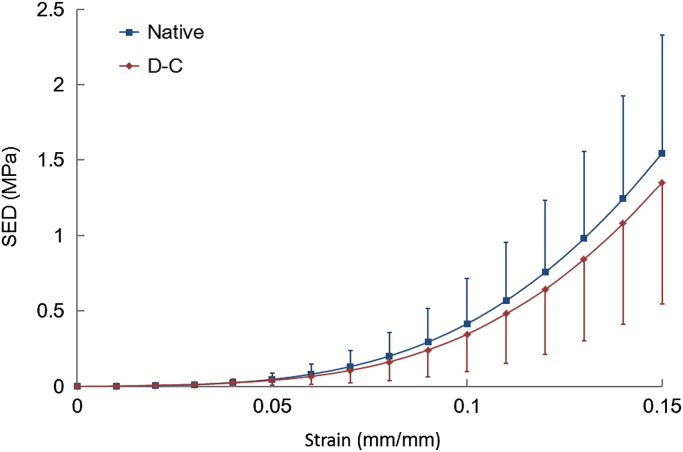
The SED calculation results as a function of strain for both native and decellularised (D-C) PT groups. Results presented as mean (*n*=8) with 95% confidence intervals. No significant difference was found between groups at any of the levels of strain investigated (Student׳s *t*-tests; *p*<0.05).

The plots of the mean strain energy density (SED) for both groups against strain are presented in Fig. 3. Very similar SED profiles were observed for both groups with no significant differences found at any of the strain locations investigated (*p*>0.05; Student׳s *t*-tests). Hence, the shape and magnitude of the stress–strain profiles were the same for native and decellularised PT groups throughout loading.

The failure mechanisms of the specimens are also highlighted in Table 1. In the native PT group there were three specimens that failed due to rupture of the tibial bone within the PMMA cement, three specimens failed due to avulsion at the tendon/bone interface and two mid-substance failures occurred. In the decellularised PT group five mid-substance failures and three tendon/bone interface avulsions occurred.

## 4 Discussion

Acellular human PT grafts have the potential to be used as a superior alternative to current autologous and allogeneic solutions in ACL replacement. They offer the availability of a product that can be refrigerated until required without tissue necrosis and biomechanical degradation, whilst eliminating the potential problems with harvesting autografts or the immunological issues of allografts. However, it is necessary to establish if the many bio-processes involved in successful decellularisation altered the mechanical properties of the tissue in an adverse manner, as some changes have been observed in similar tissues (Ingram et al., 2007, Herbert et al., 2015). Hence, this study investigated whether the decellularisation bioprocess employed by our group (Stapleton et al., 2008) altered the material properties of human PT grafts in any appreciable way. This was performed via tensile testing at a low strain rate (0.01 s^−1^) in order to negate the effects of interstitial fluid flow and focus the emphasis on the performance of the solid matrix of the material before and after decellularisation.

By employing a bi-linear model, the elastic modulus of the toe region and linear region, in addition to the transition point co-ordinates were determined for native and decellularised PT specimens. No significant differences were found between groups for any of these parameters. These same parameters were investigated in studies of patellar tendon tissues performed by Chandrashekar et al., 2012, Chandrashekar et al., 2008 who also employed a bi-linear model and found very similar values for many parameters, but lower transition strain values. The disparity between the transition points was likely due to a much slower rate of loading being used in this study. The response of human tendons at low strain has been shown to change under extension with larger strain rates, producing higher stresses for the same applied strain (Hubbard and Soutaslittle, 1984). Consequently, a higher strain rate may result in a shorter toe region and a relocation of the transition point. In fact, toe region mechanics appear more susceptible to varying strain rates than the linear region, as the linear region modulus determined in this study compared well to other studies of the patellar tendon (Blevins et al., 1994, Flahiff et al., 1995, Chandrashekar et al., 2008, Chandrashekar et al., 2012), even though these studies used higher strain rates. There are also other reports of linear region properties not changing with varying strain rates in human patellar tendon (Blevins et al., 1994), in addition to ovine flexor tendon (Lynch et al., 2003). Perhaps the key issue may be hydration, as patellar tendon has been found to be significantly more sensitive to strain rate when fully hydrated (Haut and Haut, 1997).

No significant differences were also observed between the SED profiles for both PT groups, indicating that both the energy absorption and performance of native and decellularised groups were well matched. Importantly, the range over which the SED was measured also covered the physiological range of loading these grafts would encounter if deployed in vivo (Beynnon and Fleming, 1998).

As not all specimens failed mid-substance, thus the ultimate tensile stress and strain could not be calculated. The structural performance of the specimens was examined instead by determining the load and extension at failure, with no significant differences found between native and decellularised PT groups. However, although mid-substance failure and tendon/bone avulsion were failure mechanisms present in both groups, bone rupture occurred in the native group alone (Table 1). This feature can be explained by the decellularisation process. All the marrow was removed from the bone sections of specimens in the decellularised group, which facilitated the inter-digitation of the PMMA cement with the trabecular bone, enhancing its strength and fixation. Hence, it was less likely that bone rupture would occur in the decellularised PT group during testing. Although the failure mechanisms varied, the data prior to failure was unaffected by this and the measurements of the bi-linear moduli, SED and transition points remain valid.

In a previous study, we found that decellularisation affected the elasticity of juvenile pSFT in the toe region of loading, with more extension required in acellular specimens to reach the equivalent stresses in native controls (Herbert et al., 2015). It was suggested that an alteration in the crimping patterns of the collagen fibres could explain this phenomenon. Un-crimping is a commonly used mechanism to describe the non-linear toe region behaviour of tendons and ligaments (Wang, 2006; Miller et al., 2012a, Miller et al., 2012b) and differences in crimp angles and periodicity have also been shown to generate different biomechanical load profiles in mitral valve tissue (Liao and Vesely, 2003), the patellar tendon (Stouffer et al., 1985) and gleno-humeral ligament (Bigliani et al., 1992). The exact origins of the crimping of fibres are not well understood, but it has been hypothesised that cell contraction plays a role (Herchenhan et al., 2012). Hence, if this is correct, it is intuitive that the removal of cells may alter the crimp pattern, a feature found to occur in decellularised cardiac tissue (Liao et al., 2008). Consequently, this in turn may alter the toe region biomechanics.

However, in this study, no significant difference was found in the toe region mechanics between native human PT and decellularised groups. Possible explanations for this disparity include differences in the tissues involved; particularly that the previously studied animal tissue was juvenile while the human tissue was mature. Although the effects of decellularisation (particularly with reference to the use of SDS) appear to affect some tissues and not others (Woods and Gratzer, 2005), perhaps it is the issue of tissue maturity that most warrants further consideration here. There are many microstructural changes associated with patellar tendon development and maturity including collagen crimping and crosslinking (Couppe et al., 2009, O׳brien et al., 2010) and it may be that these very changes make the tissue less susceptible to the effects of decellularisation. For example, O’ brien et al. (2010) demonstrated an increase with maturation in both the stiffness and Young׳s modulus of human patellar tendon. The increased stiffness may be due to tendon growth, however Young׳s modulus takes into account the increases in tendon length and cross-section. Hence, the changes in Young׳s modulus may be caused by age-related alterations in the underlying collagen microstructure of the tissue. Crimp angle is known to reduce with age and it has been suggested that this may be a function of changes in the cell to matrix ratio (Legerlotz et al., 2014). Hence, due to a combination of reductions in cell density and collagen crimping angle with maturity, decellularisation could have caused a reduced effect on the matrix structure and likewise the biomechanics.

Changes in HP and LP (hydroxylysyl pyridinoline and lysyl pyridinoline respectively) crosslinks are also known to contribute to the mechanical properties of tendon (Couppe et al., 2009, Willett et al., 2010, Fessel et al., 2012) and have been demonstrated to increase in response to reducing collagen density with age to maintain these properties (Couppe et al., 2009). Higher proportions of HP and LP crosslinks in mature tissue may also make the tissue more resistant to possible disruptions in the collagen matrix caused by bio-processes involved in decellularisation. Furthermore, these crosslinks are spontaneously formed from immature, chemically reducible, covalent crosslinks in the early stages of development (Patterson-Kane et al., 1997, Couppe et al., 2009). In the equine flexor tendon, there is a significant presence of the reducible crosslinks at ages of up to one year, which become HP/LP cross-links with further maturity (Patterson-Kane et al., 1997). The pSFT (obtained from animals aged 3–6 months) was also likely to have contained significant proportions of reducible cross links, possibly making it more susceptible to bio-processing effects than the mature human PT tissue investigated in this study.

There are limitations in this study worth mentioning. Firstly, strain measurement was determined using the crosshead displacement of the Instron. This assumed that the displacement experienced by the region defined by the gauge length was uniform and the same as the crosshead travel. An attempt was made to use a digital video extensometer to measure the strain, however there was difficulty in tracking the reference markers on the wet tissue. A second limitation was that although the entire PT grafts were decellularised, including the bone extremities, only the mechanical properties of the tendon element were determined. The need to cement the bone for adequate fixation precluded the possibility that this bone could be removed and separately mechanically assessed. Removal of the tendon from the bone so they can be assessed independently is also fraught with difficulties in terms of gripping the tendon specimens and fraying and separation of the collagen fibres once detached from the bone. However, future studies are planned which intend to determine the biomechanical properties of the decellularised bone components. In addition, utilising a porcine patellar tendon model, it is intended to examine the influence of maturity on collagen crimp angles and periodicity, in addition cross-linked collagen content before and after decellularisation. These graft tissues also require assessment under cyclic, physiological loading conditions to determine if significant viscoelastic changes are evident following decellularisation. Previously, our group has demonstrated that glycosaminoglycan (GAG) content is not reduced by decellularisation in porcine PT (Ingram et al., 2007). However, we intend to explore the biomechanical effect of any possible GAG reduction in acellular human PT grafts in a further study, scaling up to in-vitro implantation models to determine the dynamic moduli and the contributions of the elastic and viscous components to the grafts.

At the outset, this study aimed to test the hypothesis that the same biomechanical changes found to occur in the pSFT after decellularisation (Herbert et al., 2015) would also be present in human PT. However, this hypothesis was refuted as the toe region biomechanics of the human PT graft remained unaffected by decellularisation when tested under quasistatic loading conditions. More fundamentally, this study offers insights that mature tissue may be less susceptible to biomechanical changes caused by decellularisation bioprocesses, possibly due to age-related alterations in the collagen matrix such as crimping and crosslinking.

## Conflict of interest statement

J. Fisher and E. Ingham are consultants to and shareholders of Tissue Regenix Group plc. J. Fisher is also a consultant to DePuy Synthes, Invibio, and Simulation Solutions.

## References

[bib1] Beynnon B.D., Fleming B.C. (1998). Anterior cruciate ligament strain in-vivo: a review of previous work. J. Biomech..

[bib2] Bigliani L.U., Pollock R.G., Soslowsky L.J., Flatow E.L., Pawluk R.J., Mow V.C. (1992). Tensile properties of the inferior glenohumeral ligament. J. Orthop. Res..

[bib3] Blevins F.T., Hecker A.T., Bigler G.T., Boland A.L., Hayes W.C. (1994). The effects of donor age and strain-rate on the biomechanical properties of bone–patellar tendon–bone allografts. Am. J. Sports Med..

[bib4] Chandrashekar N., Slauterbeck J., Hashemi J. (2012). Effects of cyclic loading on the tensile properties of human patellar tendon. Knee.

[bib5] Chandrashekar N., Hashemi J., Slauterbeck J., Beynnon B.D. (2008). Low-load behaviour of the patellar tendon graft and its relevance to the biomechanics of the reconstructed knee. Clin. Biomech..

[bib6] Corry I.S., Webb J.M., Clingeleffer A.J., Pinczewski L.A. (1999). Arthroscopic reconstruction of the anterior cruciate ligament – a comparison of patellar tendon autograft and four-strand hamstring tendon autograft. Am. J. Sports Med..

[bib7] Corsetti J.R., Jackson D.W. (1996). Failure of anterior cruciate ligament reconstruction – the biologic basis. Clin. Orthop. Relat. Res..

[bib8] Couppe C., Hansen P., Kongsgaard M., Kovanen V., Suetta C., Aagaard P., Kjaer M., Magnusson S.P. (2009). Mechanical properties and collagen cross-linking of the patellar tendon in old and young men. J. Appl. Physiol..

[bib9] Crapo P.M., Gilbert T.W., Badylak S.F. (2011). An overview of tissue and whole organ decellularization processes. Biomaterials.

[bib10] Elliott D.M., Setton L.A. (2001). Anisotropic and inhomogeneous tensile behavior of the human anulus fibrosus: experimental measurement and material model predictions. J. Biomech. Eng.—Trans. ASME.

[bib11] Fessel G., Gerber C., Snedeker J.G. (2012). Potential of collagen cross-linking therapies to mediate tendon mechanical properties. J. Shoulder Elb. Surg..

[bib12] Flahiff C.M., Brooks A.T., Hollis M., Vanderschilden J.L., Nicholas R.W. (1995). Biomechanical analysis of patellar tendon allografts as a function of age. Am. J. Sports Med..

[bib13] Gilbert T.W., Sellaro T.L., Badylak S.F. (2006). Decellularization of tissues and organs. Biomaterials.

[bib14] Haut T.L., Haut R.C. (1997). The state of tissue hydration determines the strain-rate-sensitive stiffness of human patellar tendon. J. Biomech..

[bib15] Herbert A., Jones G.L., Ingham E., Fisher J. (2015). A biomechanical characterisation of acellular porcine super flexor tendons for use in anterior cruciate ligament replacement: investigation into the effects of fat reduction and bioburden reduction bioprocesses. J. Biomech..

[bib16] Herchenhan A., Kalson N.S., Holmes D.F., Hill P., Kadler K.E., Margetts L. (2012). Tenocyte contraction induces crimp formation in tendon-like tissue. Biomech. Model. Mechanobiol..

[bib17] Hubbard R.P., Soutaslittle R.W. (1984). Mechanical properties of human tendon and their age dependence. J. Biomech. Eng.—Trans. ASME.

[bib18] Ingram J.H., Korossis S., Howling G., Fisher J., Ingham E. (2007). The use of ultrasonication to aid recellularization of acellular natural tissue scaffolds for use in anterior cruciate ligament reconstruction. Tissue Eng..

[bib19] Laurencin C.T., Freeman J.W. (2005). Ligament tissue engineering: an evolutionary materials science approach. Biomaterials.

[bib20] Legerlotz K., Dorn J., Richter J., Rausch M., Leupin O. (2014). Age-dependent regulation of tendon crimp structure, cell length and gap width with strain. Acta Biomater..

[bib21] Liao J., Vesely I. (2003). A structural basis for the size-related mechanical properties of mitral valve chordae tendineae. J. Biomechan..

[bib22] Liao J., Joyce E.M., Sacks M.S. (2008). Effects of decellularization on the mechanical and structural properties of the porcine aortic valve leaflet. Biomaterials.

[bib23] Lynch H.A., Johannessen W., Wu J.P., Jawa A., Elliott D.M. (2003). Effect of fiber orientation and strain rate on the nonlinear uniaxial tensile material properties of tendon. J. Biomech. Eng.—Trans. ASME.

[bib24] Mcfarland E.G. (1993). The biology of anterior cruciate ligament reconstructions. Orthopedics.

[bib25] Miller K.S., Connizzo B.K., Feeney E., Soslowsky L.J. (2012). Characterizing local collagen fiber re-alignment and crimp behavior throughout mechanical testing in a mature mouse supraspinatus tendon model. J. Biomech..

[bib26] Miller K.S., Connizzo B.K., Feeney E., Tucker J.J., Soslowsky L.J. (2012). Examining differences in local collagen fiber crimp frequency throughout mechanical testing in a developmental mouse supraspinatus tendon model. J. Biomech. Eng.—Trans. ASME.

[bib27] O׳brien T.D., Reeves N.D., Baltzopoulos V., Jones D.A., Maganaris C.N. (2010). Mechanical properties of the patellar tendon in adults and children. J. Biomech..

[bib28] Patterson-Kane J.C., Parry D.A.D., Birch H.L., Goodship A.E., Firth E.C. (1997). An age-related study of morphology and cross-link composition of collagen fibrils in the digital flexor tendons of young thoroughbred horses. Connect. Tissue Res..

[bib29] Persson A., Fjeldsgaard K., Gjertsen J.-E., Kjellsen A.B., Engebretsen L., Hole R.M., Fevang J.M. (2014). Increased risk of revision with hamstring tendon grafts compared with patellar tendon grafts after anterior cruciate ligament reconstruction a study of 12,643 patients from the norwegian cruciate ligament registry, 2004–2012. Am. J. Sports Med..

[bib30] Petrigliano F.A., Mcallister D.R., Wu B.M. (2006). Tissue engineering for anterior cruciate ligament reconstruction: a review of current strategies. Arthroscopy—J. Arthrosc. Relat. Surg..

[bib31] Pridgen B.C., Woon C.Y.L., Kim M., Thorfinn J., Lindsey D., Hung P., Chang J. (2011). Flexor tendon tissue engineering: acellularization of human flexor tendons with preservation of biomechanical properties and biocompatibility. Tissue Eng. Part C—Methods.

[bib32] Prokopis P.M., Schepsis A.A. (1999). Allograft use in ACL reconstruction. Knee.

[bib33] Rahr-Wagner L., Thillemann T.M., Pedersen A.B., Lind M. (2014). Comparison of hamstring tendon and patellar tendon grafts in anterior cruciate ligament reconstruction in a nationwide population-based cohort study results from the danish registry of knee ligament reconstruction. Am. J. Sports Med..

[bib34] Samuelsson K., Andersson D., Karlsson J. (2009). Treatment of anterior cruciate ligament injuries with special reference to graft type and surgical technique: an assessment of randomized controlled trials. Arthroscopy—J. Arthrosc. Relat. Surg..

[bib35] Spindler K.P., Wright R.W. (2008). Anterior cruciate ligament tear. New Engl. J. Med..

[bib36] Stapleton T.W., Ingram J., Katta J., Knight R., Korossis S., Fisher J., Ingham E. (2008). Development and characterization of an acellular porcine medial meniscus for use in tissue engineering. Tissue Eng. Part A.

[bib37] Stouffer D.C., Butler D.L., Hosny D. (1985). The relationship between crimp pattern and mechanical response of human patellar tendon–bone units. J. Biomech. Eng.—Trans. ASME.

[bib38] Wang J.H.C. (2006). Mechanobiology of tendon. J. Biomech..

[bib39] Willett T.L., Labow R.S., Aldous I.G., Avery N.C., Lee J.M. (2010). Changes in collagen with aging maintain molecular stability after overload: evidence from an in vitro tendon model. J. Biomech. Eng.-Trans. Asme.

[bib40] Woo S.L.Y., Danto M.I., Ohland K.J., Lee T.Q., Newton P.O. (1990). The use of a laser micrometer system to determine the cross-sectional shape and area of ligaments – a comparative study with 2 existing methods. J. Biomech. Eng.-Trans. Asme.

[bib41] Woods T., Gratzer P.F. (2005). Effectiveness of three extraction techniques in the development of a decellularized bone-anterior cruciate ligament–bone graft. Biomaterials.

